# Nutritional characterization of the extrusion-processed micronutrient-fortified corn snacks enriched with protein and dietary fiber

**DOI:** 10.3389/fnut.2022.1062616

**Published:** 2022-12-23

**Authors:** Faiz-ul-Hassan Shah, Mian Kamran Sharif, Zulfiqar Ahmad, Adnan Amjad, Muhammad Sameem Javed, Raheel Suleman, Dur-e-Shahwar Sattar, Muhammad Amir, Muhammad Junaid Anwar

**Affiliations:** ^1^Department of Food Science and Technology, Faculty of Agriculture and Environment, The Islamia University of Bahawalpur, Bahawalpur, Punjab, Pakistan; ^2^National Institute of Food Science and Technology, University of Agriculture Faisalabad, Faisalabad, Punjab, Pakistan; ^3^Department of Human Nutrition and Dietetics, Bahauddin Zakariya University Multan, Multan, Punjab, Pakistan; ^4^Department of Food Safety and Quality Management, Bahauddin Zakariya University Multan, Multan, Punjab, Pakistan; ^5^Department of Food Science and Technology, Bahauddin Zakariya University Multan, Multan, Punjab, Pakistan

**Keywords:** extrusion, snacks, fortification, protein, dietary fiber

## Abstract

The current study focused on developing protein- and dietary fiber-enriched, micronutrient-fortified corn snacks using extrusion technology. Corn, soybean, and chickpea flour were used to develop micronutrient-fortified (Fe, Zn, I, and vitamin A, and C) extruded snacks, followed by an exploration of their nutritional traits. Soybean and chickpea were supplemented discretely (20–40/100 g) or in a combination of both (10:10, 15:15, and 20:20/100 g). According to the results, the relative proportion of the raw material composition was reflected in corn snacks' proximate composition and mineral and vitamin levels. Corn snacks with 40/100 g soy flour showed the best nutrient profile, with a maximum percent increase in protein (171.9%) and fiber (106%), as compared to the snacks developed using chickpea and/or mixed supplementation with soy and chickpea. Total dietary fiber (18.44 ± 0.34%), soluble dietary fiber (10.65 ± 0.13%), and insoluble dietary fiber (7.76 ± 0.38%) were also found to be highest in the soy-supplemented snacks (40/100 g). It was discovered that 100 g of corn snacks could provide 115–127% of the RDA for iron, 77–82% of the RDA for zinc, 90–100% of the RDA for vitamin A, and 45–50% of the RDA for vitamin C. The results for the effect of extrusion processing on amino acids showed a 2.55–45.1% reduction in essential amino acids, with cysteine and valine showing the greatest decrease and leucine and tryptophan remaining relatively stable during extrusion.

## 1. Introduction

South Asian countries are facing serious health issues because of malnutrition. The situation is worse in India, Pakistan, and Bangladesh, where half of the world's malnourished children and women reside (1). Factors such as growth and development are mainly dependent on the status of human beings. Micronutrients are required for the normal functioning of the body, including for nerve impulse conduction, for normal physical and mental function, for maintaining electrolyte balance, for regulating blood pressure, and as part of most enzymes and hormones (2). Therefore, awareness of eradicating micronutrient malnutrition has improved over the last few years (3).

Nutrition-related problems could be best addressed by food-based approaches. The use of food fortification to improve micronutrient status in a population (4, 5) without requiring any radical changes in food habits and reaching almost all sections of society (6) is one of the most effective and long-term approaches. The most important considerations are to calibrate the nutrient profile during fortification to avoid any toxicological effects, the use of multiple micronutrients, and the cost-effective addition of nutrients to foods (7).

The Composite Flour Program has been initiated by FAO, especially for the development of healthy bakery items for the masses. This initiative has paved the way to introduce an array of products with commercial significance, nutritional benefits, and additional advantages in functional and textural parameters. During the development of composite flours, factors such as consumer acceptability, nutritional quality, and functional and prophylactic benefits are considered (8). Composite flours are developed by supplementing cereal flours (rice, maize, sorghum, and pearl millet) with legume flours to alleviate prevailing malnutrition in South Asia without disturbing the quality of finished products. Despite many advancements, there are still many hurdles in preparing cereal, tuber, and legume-based formulations to replace the gluten in cereal-based products. Rice and maize as composite flours provide a better option to mimic gluten properties. Corn is an underutilized crop in Pakistan. The escalating prices of dietary staples such as wheat flour and rice in Pakistan have created an opportunity to utilize composite flour technology to produce diverse food products and to improve the economic access of the masses to food availability and security (9). In food processing, extrusion technology has been used commercially to develop shelf-stable food products. Extrusion has several advantages, including the development of the desired shape of products, the reduction of anti-nutritional factors, and improved digestibility and palatability of nutrients. During extrusion processing, the digestibility of the starch also increases due to its gelatinization (10). Corn (*Zea mays L*.) is an ideal ingredient for producing snacks through extrusion due to its starch content (11). Corn is one of the chief cash crops of Pakistan and is ranked third in importance after wheat and rice. Corn is used in various forms in the food processing industry. In developing countries, its utilization is limited to forage forms for livestock and poultry. Therefore, the production of value-added food products from corn is required.

Soybean (*Glycine max*) contains high levels of good-quality protein, making it an ideal option to increase the protein content of different food products. Soybeans contain nearly all the nutrients required for good health, including nine essential amino acids. Similarly, chickpea (*Cicer arietinum*), a good source of protein, especially after defatting, provides 25.3–28.9% protein and is a substantial source of essential fatty acids, essential amino acids, and minerals (12). Guar gum is one of the excellent sources of dietary fiber, with a high concentration of soluble dietary fiber (75%) and insoluble dietary fiber (7.6%). Physiological, structural, chemical, and technological traits of soluble and insoluble dietary fiber are different. The addition of soluble dietary fiber, which forms a gel, results in increased satiety and gastric emptying time.

The consumption of snack foods has increased with the changing lifestyle of the 21st century. Snacks are foods that are typically smaller than a regular meal and consumed between meals. In the United States, children take snacks on average six times a day. Keeping in view the increased consumption of snacks in the diet, public departments in various countries, such as Health Canada, are recommending people replace conventional snacks with healthier snacks in their diet (13).

The current study was designed to formulate corn-based micronutrient-fortified extruded snacks enriched with protein by supplementing corn with soybean, chickpea, and dietary fiber from guar gum. These snacks not only fulfill the dietary requirements of the community but also help mitigate certain micronutrient and macronutrient deficiencies in children in developing countries. Therefore, it can serve as a miracle to alleviate the malnutrition burden.

## 2. Materials and methods

### 2.1. Procurement and preparation of raw materials

For this study, corn (*Zea mays*), soybeans (*Glycine max*), chickpeas (*Cicer arietinum*), and guar gum were procured from the local market in Faisalabad, Pakistan. Micronutrient premix (Fe, Zn, I, and vitamins A and C) was provided by Fortitech Inc. A vitamin/mineral pre-mix comprising five micronutrients used for fortification (Fe as NaFeEDTA, Zn as zinc oxide, I as sodium iodide, vitamin A as retinyl palmitate, and vitamin C as ascorbic acid) was provided by Fortitech in Schenectady, New York, USA. Megazyme total dietary fiber (TDF) test kits were procured from Novozymes, Karachi, Pakistan. All reagents and standards used in the study were procured from Sigma-Aldrich (Merck KGaA, Darmstadt, Germany). Physical impurities were removed with manual cleaning. Dehulling and milling of both soybeans and chickpeas were carried out to get flour, followed by defatting with the solvent method. Defatted soy and chickpea flours were stored in separate polythene bags with the corn flour at 25°C for further use.

### 2.2. Analysis of the raw materials

#### 2.1.1. Proximate composition

Corn, soybean, and chickpea flours were analyzed for crude fat (AACC Method No. 30-25), crude protein (AACC method no. 46-10), moisture (AACC Method No. 46-30), crude fiber (AACC method no. 32-10), and total ash (AACC method no. 08-01) by the respective methods described by AACC (14).

#### 2.2.2. Mineral content

Raw materials (corn, soybean, and chickpea flours) were analyzed for a mineral profile by wet digestion according to the method given in AOAC (15). The sample (0.5) was first digested at low temperature (60–70°C) with 10 mL of HNO_3_ for 20 min in a 100-mL conical flask on a hot plate. Then, it was digested at a high temperature (190°C) with 5 mL of concentrated HClO_4_ until the contents of the flask became clear. The mineral contents of the samples were determined using the respective standard curves prepared for each element. Aliquots were used to estimate Na and K by a flame photometer (Sherwood Flame Photometer, Cambridge, UK). Sodium and potassium contents were determined by using the flame photometer 410 (Sherwood Scientific Ltd., Cambridge, UK), whereas magnesium, calcium, iodine, iron, and zinc contents were determined through an atomic absorption spectrophotometer (Varian AA240, Varian Medical Systems Australasia Ltd., Belrose, Australia). The samples were quantified against standard solutions of known concentrations that were analyzed concurrently.

#### 2.2.3. Analysis of vitamins

AOAC, method no. 2012.09, was used for the determination of the vitamin A content of flour samples (15). In contrast, Hernández et al. (16) method was used for vitamin C determination.

##### 2.2.3.1. Vitamin A

Stock solutions (100 μg/mL) of retinyl palmitate were separately prepared in 100 mL of absolute ethanol. The working standard (5 μg of retinyl palmitate/mL) was prepared by diluting 5 mL of retinyl palmitate stock solution to 100 mL with ethanol. The concentration of the working standard was determined by measuring absorbance at 328 nm and dividing it by the specific absorption coefficient E (1%/1 cm) of 975 for retinyl palmitate. Calibration was performed using a concentration range of 0.09–2.0 μg/mL.

Samples were diluted with hexane (1:10), and 200 mL of the sample was transferred to a screw-capped tube, followed by the addition of 600 mL of methanol. The solution was vortex mixed, followed by centrifugation (3,000 g, 5 min) and filtration through a 0.45 mm pore-size filter, and the aliquot was injected into the HPLC (17).

The HPLC system (Perkin Elmer-200 Series, PerkinElmer Life, and Analytical Sciences, USA) was used in this study. HPLC was assisted with a degasser, an auto-sampler, a binary pump, and a UV/Vis detector. A reversed-phase C_18_ column (150 × 4.6 mm, 3.5 μm particle sizes) was used. Total Chrome Software was used to evaluate and quantify. Acetonitrile/methanol (75:25%) was used as the mobile phase, which was delivered at a 1.0 mL/min flow rate and held at 40°C.

The sample volume to be injected into HPLC was 20 μL with the (96:4) mobile phase consisting of methanol and water; elution was performed at a flow rate of 2 mL/min. The temperature of the analytical column was 45°C. Vitamin A was detected at 265 and 325 nm for 15 min. The quantification was performed using the retention times and peak areas of the standards and samples.

##### 2.2.3.2. Vitamin C

Then, 0.25 g of DCIP was dissolved in 500 mL of distilled water to prepare the 2,6-dichloroindophenol (DCIP) solution, followed by the addition of sodium bicarbonate (0.21 g). The final volume of the solution was made up to 1 L with the help of distilled water. The concentration of the DCIP was almost 250 mg of DCIP/L. The next step was the standardization of the DCIP solution. Subsequently, 5 mL of an ascorbic acid solution was carefully pipetted into a 250-mL Erlenmeyer flask. The concentration of standard ascorbic acid was recorded. Afterward, 2 mL of the sulfuric acid mixture was added, and about 25 mL of distilled water was added to the flask. The flask was swirled to mix the solution. Then, 50 mL of the DCIP solution was added to the burette. DCIP was used to titrate the ascorbic acid until a permanent light red or pink color appeared, which lasted more than 30 s. After standardization, a standard DCIP solution was used to titrate the sample until a permanent light red or pink appeared, which lasted for more than 30 s. Vitamin C was calculated by the oxidation balance (16).

### 2.3. Extrusion formulations

As shown in Table 1, different levels of soybean and chickpea flour were used for the preparation of extrusion formulations. All formulations contained 7 g of guar gum per 100 g, except Control 1 treatment. In all treatments, minor ingredients (table salt, distilled mono-glycerides, and lecithin) were added, along with a micronutrient premix (Fe, Zn, I, and vitamins A and C) as per 3 g of the RDA per 100 g of the treatment formulations.

**Table 1:** Extrusion formulations (g/100 g).

Treatments^*^	**Guar gum**	**Corn flour**	**Soy flour**	**Chickpea flour**
Control 1	–	97	–	–
Control 2	7	90	–	–
T_1_	7	70	20	–
T_2_	7	60	30	–
T_3_	7	50	40	–
T_4_	7	70	–	20
T_5_	7	60	–	30
T_6_	7	50	–	40
T_7_	7	70	10	10
T_8_	7	60	15	15
T_9_	7	50	20	20

* The remaining formulation (3/100 g) was distilled mono-glycerides, table salt, and lecithin along with micronutrient premix comprising nutrients according to the percent daily value of added nutrients.

### 2.4. Extrusion processing

A pilot-scale single screw extruder (DGP-50, Henan Manufacturer, China) with instrumental specifications, i.e., a feed rate of 30 kg/h, a barrel diameter of 50 mm, and a temperature of 120–180°C, was used. The temperature was maintained at 150°C during the processing of corn snacks with the help of water circulation and monitored with the help of a thermocouple. The die was fitted with one circular insert of 4.2 mm diameter × 18.90 mm length. The operating variable was adjusted with the help of a pre-run of the extruder so that products with desired physical and textural properties were obtained. As the product exited the extruder barrel, it was collected in the trays and cooled at room temperature, followed by the collection of samples in the zip-lock polythene bags for further analysis.

### 2.5. Chemical composition of micronutrient-fortified corn snacks

Proximate composition, mineral content, and vitamins A and C were determined by following the methods described earlier.

Megazyme Test Kit was used to determine the dietary fiber content of corn snacks using the AACC (Method 32-05) and AOAC (Method 985.29) methods. The details of the procedures are given as follows:

#### 2.5.1. Total dietary fiber (TDF)

The samples were dispersed in a buffer solution and incubated with heat-stable α-amylase at a temperature of 95–100°C for 35 min. After cooling the samples to 60°C, 100 l of protease solution was added and incubated at 60°C for 30 min. Finally, these contents were incubated with amyloglucosidase at 60°C for 30 minutes. The fiber contents were precipitated by the addition of alcohol in 1:4 ratio. The contents were filtered and washed with alcohol and acetone. A blank was run through the entire procedure along with test samples to calculate any contribution from reagents to the residue.

#### 2.5.2. Soluble dietary fiber (SDF)

The samples were dispersed in a buffer solution and incubated with heat-stable α-amylase at 95–100°C for 35 min. After cooling the samples to 60°C, 100 μL of protease solution was added, and the contents were incubated at 60°C for 30 min. Finally, the contents by adding amyloglucosidase were incubated at a temperature of 60°C for 30 min. The residue after filtration was washed and rinsed with 10 mL of water. The filtrate and water washing were weighed, and soluble dietary fiber was precipitated with four volumes of ethyl alcohol. The contents were filtered and dried, and corrected for ash and protein contents. A blank was also run simultaneously through the entire procedure along with test samples to calculate any contribution from reagents to the residue.

#### 2.5.3. In-soluble dietary fiber (IDF)

The samples were dispersed in a buffer solution and incubated with heat-stable α-amylase at a temperature of 95–100°C for 35 min. After cooling up to 60°C, the samples were incubated by adding 100 μL protease solutions at 60°C for 30 min, and then the contents were incubated by adding amyloglucosidase at 60°C for 30 min. The residue after filtration was washed and rinsed with 10 mL of water. The resultant residue was weighed, and soluble dietary fiber was precipitated with four volumes of ethyl alcohol. The contents were filtered, dried, and corrected for ash and protein contents. A blank was also run simultaneously through the entire procedure to calculate any contribution from reagents to the residue.

#### 2.5.4. Calorific value

The calorific value of the extruded snack was determined by using an oxygen bomb calorimeter (C2000 Basic, IKA-WERKE, Germany), as described by 9. The sample (0.5 g) was taken into the metallic decomposition vial. The vial was unscrewed and fastened by a cotton thread with a loop onto the middle of the ignition wire before loading the sample. Then, the screw cap was tightened. The decomposition vial was guided into the filler head through the open measuring cell cover until it was in place. The start button was pushed, and the measuring cell cover was closed. An electric spark was used to burn the sample contained within the vial. The heat produced was noted by C5040 CalWin software of the computer (IKA-Werke, Germany) and displayed as calories per gram of a sample.

### 2.6. Amino acids profile

The amino acid profile of the raw materials was assessed to calculate the relative amino acid concentration in the extrusion formulations as per proportion in the final formulation treatments, followed by the evaluation of the fortified corn snacks for the essential and non-essential amino acids. The amino acid composition of the extrusion formulations and extruded snacks was used to estimate the impact of extrusion processing on the amino acid profile in extruded fortified corn snacks. The amino acid profile of the snacks was determined through an automatic amino acid analyzer (Hitachi L8500, Tokyo, Japan) by following the method described by Adeyeye and Afolabi (18). Then, 30 mg of defatted ground sample, 5 μm of leucine, and 5 mL of 6 M HCl were filled in a glass ampule. Evacuation of the ampule was done using liquid nitrogen, followed by ampule sealing with a burner. The hydrolyzation of the ampoules was done in an oven at 110°C for 24 h. The tip of the ampoule was broken down to cool and filter the contents in it. The contents were dried in a rotary evaporator at 40°C under a vacuum. Acetate buffer (pH 2.2) was used for the preparation of samples for different amino acids. Subsequently, 5 μL of acetate buffer was used for neutral amino acids and 10 μL for basic amino acids. The resultant sample solution was dispensed into the amino acid analyzer cartridge. A peak area comparison of the standards and samples was made for the quantification of the amino acid content.

### 2.7. Statistical analysis

The collected data were subjected to statistical analysis using SPSS version 25. For the quantitative variables, frequency, percentages, and means (standard deviation) were used. An analysis of variance (ANOVA) was performed to determine the significance of the treatments used in the study. Tukey's honest significance test was used for posthoc analysis. The level of significance was taken as a *p*-value of ≤ 0.005 (19).

## 3. Results and discussion

Protein- and dietary fiber-enriched, nutrient-dense extruded snacks were developed using soy, chickpea, and corn flours, along with micronutrient fortification. Soybean, chickpea, and corn flours were subjected to chemical and nutritional analyses for the estimation of nutrient potential. The extruded snacks were studied for nutritional traits such as proximate composition, vitamins, minerals, amino acids, dietary fiber, and caloric evaluation.

### 3.1. Chemical composition of raw materials

The results of the proximate composition of raw materials (corn, soy, and chickpea flours) showed the highest moisture content (10.61 ± 0.21%) in the corn flour and the lowest in the soy flour (7.20 ± 0.27%). Soy flour had the highest protein level (46.2 ± 0.17%), followed by chickpea flour (22.5 ± 0.11%). Soy flour was subjected to defatting before use, which is why crude fat was lowest (1.22 ± 0.13%) in defatted soy flour, followed by corn flour (3.86 ± 0.07%) and chickpea flour (6.51 ± 0.12%). The crude fiber of the chickpea flour was found to be the highest (17.5 ± 0.08%) among all raw materials. Ash content was highest (3.06 ± 0.03%) in soy flour among all the raw materials.

Mineral analysis depicted that corn and chickpeas were excellent sources of sodium, i.e., 57.3 ± 4.2 mg/100 g and 64 ± 5.4 mg/100 g, respectively. Soybean showed a relatively low sodium content (20 ± 1.5 mg/100 g). The highest levels of potassium, calcium, and magnesium (2,384 ± 48.2 mg/100 g, 241 ± 25.3 mg/100 g, and 290 ± 12.3 mg/100 g, respectively) were found to be in soybeans. Zinc and iron contents, which ranged from 1.73 ± 0.10 to 2.81 ± 0.14 mg/100 g and 2.38 ± 0.64 to 9.24 ± 0.71 mg/100 g, respectively, were far below the daily requirements, which highlighted the need for the fortification of the food products containing corn, soybean, and chickpea as chief ingredients.

Corn showed maximum vitamin A content (214 ± 5.3IU), followed by chickpea (41 ± 2.2IU) and soy (40 ± 3.1IU). It is worth mentioning that none of the raw materials contained vitamin C. It was concluded from the results that the fortification of vitamin C would be obligatory to fulfill nutrient requirements in the diet. The results for proximate composition, mineral composition, and vitamin composition were consistent with earlier findings (20–22).

### 3.2. Compositional and nutritional analysis of micronutrient-fortified corn snacks

**Figure 1:**
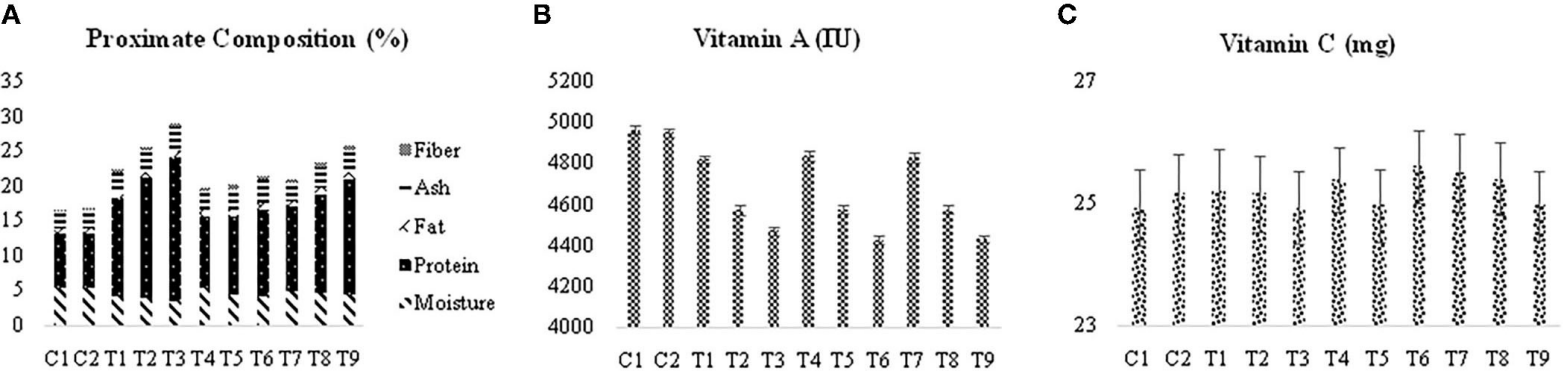
**(A)** Means for the effect of treatments on the proximate composition (%). **(B)** Means for the effect of treatments on the vitamin A content, **(C)** means for the effect of treatments on the vitamin C content. C1, control 1; C2, control 2; T1, corn snacks supplemented with 20/100 g of soy flour; T2, corn snacks supplemented with 30/100 g of soy flour; T3, corn snacks supplemented with 40/100 g of soy flour; T4, corn snacks supplemented with 20/100 g of chickpea flour; T5, corn snacks supplemented with 30/100 g of chickpea flour; T6, corn snacks supplemented with 40/100 g of chickpea flour; T7, corn snacks supplemented with 10:10/100 g of soy and chickpea flour; T8, corn snacks supplemented with 15:15/100 g of soy and chickpea flour; T9, corn snacks supplemented with 20:20/100 g of soy and chickpea flour.

Significant differences were observed for the crude protein, moisture, crude fat, crude fiber, and ash content in fortified corn snacks (Figure 1). It was noted that the lowest moisture level (3.40 ± 0.02%) was present in corn snacks developed with 40/100 g of soy flour supplementation. Corn snacks developed without supplementation had a high moisture content (5.400.01%). It can be concluded from the results that the supplementation with soy and chickpea flours resulted in decreased moisture content in the corn snacks. Varied moisture content (7.1 ± 0.17 to 9.1 ± 0.34%) in extruded snacks prepared using corn and rice was reported in another study as well (23). Means for crude protein content (7.600.10%) were lowest in corn snacks extruded without soy or chickpea flour supplementation. The maximum value (20.67 ± 0.46%) of proteins was noticed in corn snacks developed with 40/100 g of soy flour supplementation, while the lowest value (14.0 ± 0.50%) was noted in corn snacks developed with 20/100 g of soy flour supplementation. It was also concluded from the results that the protein contents of soy-supplemented corn snacks were higher than those of chickpea-supplemented snacks at each supplementation level.

A minimum amount of crude fat (0.79 ± 0.01%) was found in non-supplemented corn snacks. An increase in crude fat was observed with increasing supplementation levels, and corn snacks supplemented with chickpea flour showed higher crude fat content as compared to both the soy-supplemented and mixed-supplemented snacks.

Ash content was lowest (2.50 ± 0.03%) in the non-supplemented corn snacks, followed by corn snacks that contained only 7/100 g of guar gum (2.64 ± 0.01%). Soy flour-based snacks had the highest ash content (3.390.02%) of any supplemented snack. It was also observed that the ash of soy-supplemented corn snacks was higher than that of chickpea-supplemented corn snacks at each level of supplementation.

The proximate composition of the corn snacks revealed that the non-supplemented corn snacks contained the least amount of crude fiber (0.300.02%). Soy-supplemented corn snacks depicted the highest amount of crude fiber content as compared to others due to escalated levels of supplementation.

The results of the present research cohere with a previous study on the development of crisp from soy flour (25–40/100 g) and rice flour by supercritical fluid extrusion. Protein (334–568%) and dietary fiber (571–901%) were improved in the final products due to soy flour supplementation (24). Another study suggested that the incorporation of soy flour into cereals can assist in overcoming the deficiency of the protein (25). Similarly, in a study, a novel source of protein, i.e., spirulina, was used to increase the protein content of dry pasta, with the amount of protein ranging from 12.91/100 to 23.49/100 g in the final products (26). Another study concluded that protein-rich (40–60/100 g dry basis) extruded products prepared from a soy protein isolate-corn flour blend can be prepared with minimum moisture, ranging from 2.2 to 3.5% (11).

### 3.3. Mineral profile

Fortified corn snacks showed significant differences in calcium, potassium, magnesium, sodium, iron, and zinc content in comparison with the control (Table 2). The maximum value (100.70 ± 9.21 mg/100 g) of calcium was observed in corn snacks containing 40/100 g of soy flour, whereas the least value (6.62 ± 0.59 mg/100 g) was observed in corn snacks extruded without soy or chickpea flour supplementation.

**Table 2:** Means for the effect of treatments on the mineral content (mg/100 g), dietary fiber (g/100 g), and calories (K cal/g) of extruded corn snacks.

**Treatments**	**Ca**	**K**	**Mg**	**Na**	**Fe**	**Zn**	**Total dietary fiber**	**Soluble dietary fiber**	**Insoluble dietary fiber**	**Calorific value**
C1	6.62 ± 0.59i	296.88 ± 16.64i	88.36 ± 5.54f	4.71 ± 0.21j	10.95 ± 0.89c	10.65 ± 0.43c	10.36 ± 0.43c	4.56 ± 0.21e	5.80 ± 0.21c	3,933 ± 4.22f
C2	6.65 ± 0.53i	297.27 ± 18.94i	89.93 ± 6.35f	4.73 ± 0.82j	10.27 ± 1.32b	10.59 ± 0.64c	15.40 ± 0.67b	8.33 ± 0.17d	6.36 ± 0.31bc	3,910 ± 3.68g
T1	53.69 ± 4.21d	710.50 ± 25.32d	129.60 ± 8.53c	7.67 ± 0.74i	15.49 ± 1.01ab	10.78 ± 0.64bc	17.12 ± 0.63ab	10.03 ± 0.32abc	7.05 ± 0.12ab	4,009 ± 6.43e
T2	76.58 ± 6.32b	913.84 ± 30.67b	148.67 ± 7.52b	9.25 ± 0.22h	15.98 ± 1.32ab	10.86 ± 0.86abc	17.78 ± 0.56ab	10.34 ± 0.22ab	7.41 ± 0.25ab	4,030 ± 4.87d
T3	100.70 ± 9.21a	1,124.4 ± 55.32a	166.92 ± 4.98a	10.69 ± 0.82g	17.03 ± 1.56a	10.92 ± 0.94abc	18.44 ± 0.34a	10.65 ± 0.13a	7.76 ± 0.38a	4,064 ± 6.31a
T4	14.41 ± 1.32h	402.82 ± 34.21h	102.98 ± 9.53e	16.57 ± 1.39d	14.47 ± 0.93ab	11.02 ± 0.91abc	15.50 ± 0.61b	8.60 ± 0.42cd	6.87 ± 0.19abc	4,043 ± 5.35c
T5	18.04g ± 1.42h	456.50 ± 29.54g	110.23 ± 10.01de	22.44 ± 1.11b	15.05 ± 0.78ab	11.23 ± 0.65ab	15.60 ± 0.52b	8.87 ± 0.34bcd	6.70 ± 0.21abc	4,049 ± 4.62bc
T6	21.93 ± 2.03g	510.88 ± 40.23f	117.56 ± 9.32d	28.35 ± 1.23a	15.31 ± 1.11ab	11.33 ± 0.34a	15.77 ± 0.53ab	9.41 ± 0.13abcd	7.04 ± 0.15ab	4,061 ± 3.53ab
T7	33.27 ± 3.12f	558.97 ± 49.32e	115.04 ± 10.65d	12.14 ± 1.76f	14.65 ± 1.12ab	10.80 ± 1.02bc	16.36 ± 0.61ab	9.45 ± 0.21abcd	6.88 ± 0.20abc	4,005 ± 6.72e
T8	47.84 ± 3.89e	688.33 ± 54.34d	128.51 ± 9.43c	15.82 ± 0.98e	15.22 ± 1.26ab	10.85 ± 0.53bc	16.64 ± 0.48ab	9.47 ± 0.18abcd	7.14 ± 0.17ab	4,015 ± 5.98e
T9	61.04 ± 5.87c	816.55 ± 38.2c	142.42 ± 12.76b	19.51 ± 0.78c	15.64 ± 1.43ab	10.95 ± 0.32abc	16.92 ± 0.58ab	9.49 ± 0.17abcd	7.40 ± 0.19ab	4,030 ± 3.47d

The means carrying the same letter in a column differed non-significantly (p > 0.05).

C1, control 1; C2, control 2; T1, corn snacks supplemented with 20/100 g of soy flour; T2, corn snacks supplemented with 30/100 g of soy flour; T3, corn snacks supplemented with 40/100 g of soy flour; T4, corn snacks supplemented with 20/100 g of chickpea flour; T5, corn snacks supplemented with 30/100 g of chickpea flour; T6, corn snacks supplemented with 40/100 g of chickpea flour; T7, Corn snacks supplemented with 10:10/100 g of soy and chickpea flour; T8, corn snacks supplemented with 15:15/100 g of soy and chickpea flour; T9, Corn snacks supplemented with 20:20/100 g of soy and chickpea flour.

Corn snacks showed potassium levels ranging from 402.82 ± 34.21 to 1,124.4 ± 55.32 mg/100 g in the snacks that contained soy and chickpeas. In contrast, non-supplemented corn snacks contained 296.88 ± 16.64 mg/100 g potassium. An analysis of treatment supplemented with soy or chickpea flour for magnesium content revealed a range of 102.98 ± 9.53 to 166.92 ± 4.98 mg/100 g among all the soy or chickpea flour-supplemented snacks. However, sodium content ranged from 7.67 ± 0.74 to 28.35 ± 1.23 mg/100 g among the soy- and chickpea-supplemented treatments. Non-supplemented corn snacks showed the lowest sodium content (4.71 ± 0.21 mg/100 g), whereas the highest sodium content (28.35 ± 1.23 mg/100 g) was noted in corn snacks containing 40/100 g of chickpea flour.

Soy and chickpea-supplemented corn snacks showed iron content ranging from 14.47 ± 0.93 to 15.98 mg/100 g. In contrast, non-supplementation snacks contained 10.95 ± 0.89 mg/100 g of iron. It was also noted that the iron level decreased to 10.27 ± 1.32 mg/100 g with the addition of guar gum. Zinc was found to be more abundant in the snacks supplemented with chickpea flour as compared to soy flour, with the highest content of this nutrient (11.33 ± 0.34 mg/100 g) in snacks made with 40/100 g of chickpea flour. However, a minimum (10.65 ± 0.43 mg/100 g) level of zinc was noted in the non-supplemented snacks.

Existing literature suggests that the bioavailability of the minerals improves during extrusion processing owing to the inactivation of anti-nutritional factors, e.g., phytates (24, 27). Moreover, instead of harsh processing conditions, mineral content remains steady during extrusion, and nearly no mineral loss occurs. Some studies even observed an increase in iron, which is linked to the liberation of iron from complex molecules that increases its bioavailability (27). Similarly, another study that used superficial fluid extrusion to prepare micronutrient-fortified rice soy crisp found iron content ranging from 26.19 to 32.09 mg/100 g and zinc content ranging from 13.65 to 14.92 mg/100 g. The study revealed 100% retention of all added minerals, with even a 25% increase in iron level in the crisps (24). Another study revealed that 10% of the RDA of Zn can be filled by consuming spirulina-supplemented corn crisps (28).

### 3.4. Vitamin A and C content

Vitamin A, the content of the corn snacks, was observed at its maximum (4,962.0 ± 20.12IU) in the non-supplemented corn snacks, followed by the corn snacks (4,950.5 ± 19.32IU) containing 7 g/100 of guar gum (Figure 1). It was also noted that the lowest levels of soy, chickpeas, and mixed supplementation with soy and chickpea contain 4,820.2 ± 17.43, 4,834.5 ± 23.42, and 4,825.5 ± 23.82 IU vitamin A, respectively. Corn is the best source of vitamin A among all the raw materials, as shown by the composition of the raw materials. As a result, corn-based snacks have a higher level of vitamin A. No statistical difference was observed regarding mean vitamin C content. This indicates that supplementation with soy and chickpea has no effect on the vitamin C content of the extruded snacks. The only source of vitamin C is the fortification of snacks with ascorbic acid.

The nutritional composition of the extruded product is mainly dependent on the processing variables of extrusion (29). Some studies utilizing novel extrusion processes retain about 50% of vitamins A and C at low-shear and low-temperature conditions (24). Similarly, 64–76% retention of vitamin C and 55–58% retention of vitamin A were noted in puffed rice made using supercritical fluid extrusion (27).

### 3.5. Dietary fiber

Dietary fiber in the corn snacks developed with 40/100 g of soy flour showed a maximum level of 18.44 ± 0.34%, whereas a minimum level of 10.36 ± 0.43% was observed in the non-supplemented corn snacks (Table 2). The total dietary fiber of the corn snacks containing soy flour was higher at each supplementation level as compared to the other snacks. The study conducted by Alonso et al. (30) and Varo showed an insignificant variation in total dietary fiber in the product extruded at 161–180°C. Although extrusion showed insignificant changes in the total dietary fiber, the distribution of the soluble to insoluble dietary fiber changed during extrusion (31).

Overall, soluble dietary fiber ranged from 9.41 ± 0.13 to 10.65 ± 0.13%. It was observed that dietary fiber increased with increased supplementation. Soy-supplemented corn snacks have more soluble dietary fiber as compared to the other treatments. Onwulata et al. (32) revealed a 10% increase in soluble dietary fiber after the extrusion of the food product.

As depicted in Table 2, the means for the insoluble dietary fiber of corn snacks showed that insoluble dietary fiber was lowest (5.80 ± 0.21%) in the non-supplemented corn snacks, followed by corn snacks containing guar gum along with corn (6.36 ± 0.31%). The insoluble dietary fiber of the soy-supplemented corn snacks was higher than that of the remaining corn snacks at each supplementation level. Insoluble dietary fiber reduction is linked with extrusion processing, as presented in a study conducted on the extrusion of black beans and cereals (31).

### 3.6. Calorific value

The corn snack containing 40/100 g soy flour had the highest mean calorific value (40,646.31 kcal/g). The minimum calorific value (3,933 ± 4.22 kcal/g) was noted in non-supplemented corn snacks. Overall, soy-supplemented corn snacks showed higher calorific values as compared to chickpea-supplemented snacks (Table 2).

### 3.7. Effect of extrusion on the amino acid composition of fortified corn snacks

**Figure 2:**
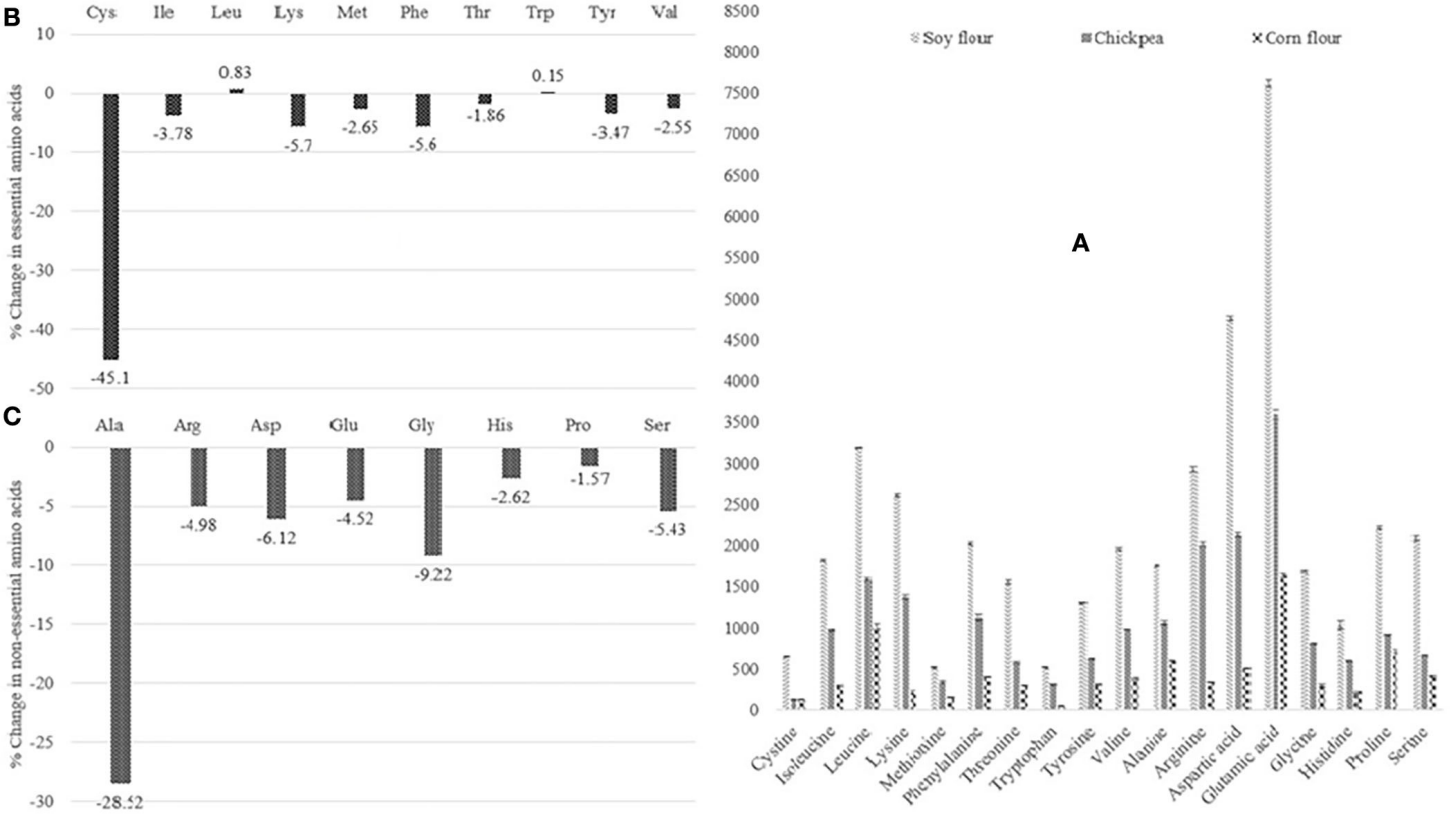
**(A)** Means for the amino acid profile of raw materials [x-axis, amino acids; Y-axis, concentration (mg/100 g)]; **(B)** Effect of extrusion processing on the essential amino acid of extruded corn snacks (x-axis, essential amino acids; Y-axis, % change in essential amino acid); **(C)** Effect of extrusion on the non-essential amino acid of extruded corn snacks (x-axis, non-essential amino acids; Y-axis, % change in non-essential amino acids).

The basic aim behind supplementing soy and chickpeas with corn was to improve the quantity and quality of protein in the final extruded product. Extrusion has both positive and negative effects on different amino acids. In the current study, first, raw materials, i.e., soy, chickpea, and corn flour, were analyzed for the amino acid profile. The results are summarized in Figure 2. These concentrations of the amino acids were used to compute the amino acid profile of the extrusion premixes based on their proportion in the final formulation. The amino acid profile was evaluated using the essential and non-essential amino acid composition (mg/100 g) of extruded corn snacks based on the raw material composition. The results of the analysis are shown in Table 3. The means for the essential and non-essential amino acids (mg/100 g) of extruded fortified corn snacks are shown in Table 4.

**Table 3:** Amino acids composition (mg/100g) of extrusion formulation of corn snacks based on raw material composition.

**Treatments**	**Essential amino acids**								
	**Cysteine**	**Isoleucine**	**Lucien**	**Lysine**	**Methionine**	**Phenylalanine**	**Threonine**	**Tryptophan**	**Tyrosine**
C1	124.16 ± 8.41f	285.18 ± 3.22j	979.70 ± 9.15j	211.46 ± 5.33i	149.38 ± 3.24g	397.70 ± 3.87j	285.18 ± 2.47j	49.47 ± 2.43i	372.48 ± 5.46j
C2	115.20 ± 6.91g	264.60 ± o5.95k	909.43 ± 7.17k	196.20 ± 8.34j	138.60 ± 8.72h	369.23 ± 4.35k	264.60 ± 9.60kS	45.90 ± 1.78j	345.60 ± 6.54k
T1	220.23 ± 4.44c	569.80 ± 8.51e	1,345.67 ± 8.53d	674.60 ± 5.85d	212.45 ± 8.90d	691.43 ± 9.07e	517.87 ± 4.67d	139.96 ± 4.65f	660.83 ± 7.85e
T2	272.40 ± 2.12b	722.40 ± 8.90b	1,563.45 ± 9.35b	913.80 ± 5.13a	248.72 ± 5.63b	852.12 ± 7.25b	644.44 ± 5.83b	186.91 ± 2.46c	818.41 ± 9.35b
T3	324.80 ± 8.72a	875.33 ± 7.55a	1,781.43 ± 9.80a	115.34 ± 9.24k	285.40 ± 7.50a	1,013.45 ± 4.53a	771.34 ± 4.84a	233.92 ± 8.72a	976.23 ± 8.39a
T4	114.35 ± 3.82h	400.23 ± 6.20i	1,026.05 ± 8.20i	427.55 ± 5.42h	176.20 ± 6.17f	512.32 ± 5.35i	321.45 ± 7.10i	96.90 ± 1.57h	463.65 ± 7.54i
T5	113.95 ± 3.24h	468.34 ± 4.60h	1,084.75 ± 9.14h	543.22 ± 7.17g	195.34 ± 0.08e	583.50 ± 4.97h	349.87 ± 9.35h	122.44 ± 2.46g	522.65 ± 4.14h
T6	113.53 ± 2.40h	535.85 ± 5.67f	1,143.12 ± 7.18g	658.90 ± 3.95e	213.85 ± 2.50d	655.23 ± 6.35f	378.33 ± 8.34g	147.92 ± 3.75e	581.70 ± 5.20f
T7	167.17 ± 2.65e	485.13 ± 5.18g	1,185.52 ± 4.60f	551.07 ± 4.40f	194.13 ± 3.58	601.50 ± 8.93g	419.62 ± 2.54f	118.42 ± 1.45h	562.25 ± 4.80g
T8	193.16 ± 2.44d	595.24 ± 7.70d	1,323.78 ± 9.80e	728.51 ± 5.20c	221.85 ± 2.98c	717.75 ± 8.56d	497.13 ± 5.30e	154.65 ± 3.53d	670.53 ± 4.35d
T9	219.15 ± 5.23c	705.42 ± 2.48c	1,462.05 ± 2.40c	905.95 ± 9.15b	249.62 ± 5.72b	834.42 ± 5.20c	574.65 ± 2.69c	190.92 ± 3.24b	778.85 ± 3.53c
**Treatments**	**Non-Essential amino acids**							
	**Alanine**	**Arginine**	**Aspartic acid**	**Glutamic acid**	**Glycine**	**Histidine**	**Proline**	**Serine**
C1	583.94 ± 7.64j	323.01 ± 3.20j	496.64 ± 3.95j	1,600.5 ± 6.11h	285.18 ± 2.23i	211.46 ± 3.25i	695.49 ± 2.53j	397.70 ± 7.43j
C2	541.80 ± 7.25k	299.70 ± 2.24k	460.80 ± 2.51k	1,485.78 ± 5.32i	264.60 ± 3.76j	196.20 ± 1.41j	645.30 ± 5.83k	369.53 ± 8.75k
T1	773.46 ± 6.94e	819.10 ± 5.42f	1,310.48 ± 9.35e	2,679.64 ± 10.2d	543.80 ± 6.28d	360.68 ± 3.24d	945.92 ± 4.97d	705.34 ± 4.34d
T2	889.25 ± 3.20b	1,078.87 ± 6.17c	1,735.20 ± 8.42b	3,276.78 ± 20.2b	683.40 ± 7.21b	442.86 ± 2.99b	1,096.21 ± 8.22b	873.24 ± 3.20b
T3	1,005.55 ± 9.74a	1,338.55 ± 5.35a	2,160.56 ± 9.42a	3,873.78 ± 18.1a	823.64 ± 6.42a	525.46 ± 5.32a	1,246.52 ± 9.30a	1,041.76 ± 5.42a
T4	633.35 ± 4.54i	636.34 ± 5.42i	785.45 ± 5.21i	1,873.65 ± 10.2g	367.35 ± 2.18h	273.22 ± 2.87h	685.05 ± 6.32i	420.20 ± 6.11i
T5	679.13 ± 4.23h	804.63 ± 5.20g	947.17 ± 8.01h	2,067.98 ± 9.81f	418.73 ± 5.32g	311.74 ± 3.64g	704.93 ± 7.17h	445.83 ± 5.23h
T6	724.95 ± 7.62f	972.95 ± 8.56d	1,109.27 ± 9.98f	2,262.30 ± 10.4e	470.10 ± 4.37e	350.22 ± 2.34e	724.80 ± 4.22g	471.42 ± 8.37g
T7	703.38 ± 5.51g	727.77 ± 8.93h	1,047.74 ± 8.29g	2,276.33 ± 9.81e	455.58 ± 4.99f	316.94 ± 5.10f	815.48 ± 7.32f	562.61 ± 7.42f
T8	784.16 ±± 8.23d	941.78 ± 6.35e	1,341.15 ± 9.13d	2,671.99 ± 10.9d	551.06 ± 6.98d	377.25 ± 4.26c	900.56 ± 6.91e	659.42 ± 5.45e
T9	864.95 ± 4.12c	1,155.70 ± 4.97b	1,634.63 ± 7.99c	3,067.65 ± 12.2c	646.55 ± 5.62c	437.64 ± 3.53b	985.65 ± 7.77c	756.20 ± 6.98c

The means carrying the same letter in a column differed non-significantly (p > 0.05).

C1, control 1; C2, control 2; T1, corn snacks supplemented with 20/100 g of soy flour; T2, corn snacks supplemented with 30/100 g of soy flour; T3, corn snacks supplemented with 40/100 g of soy flour; T4, corn snacks supplemented with 20/100 g of chickpea flour; T5, corn snacks supplemented with 30/100 g of chickpea flour; T6, corn snacks supplemented with 40/100 g of chickpea flour; T7, corn snacks supplemented with 10:10/100 g of soy and chickpea flour; T8, corn snacks supplemented with 15:15/100 g of soy and chickpea flour; T9, corn snacks supplemented with 20:20/100 g of soy and chickpea flour.

**Table 4:** Means for the effect of treatments on essential and non-essential amino acids (mg/100g) of extruded corn snacks.

**Treatments**	**Cysteine**	**Isoleucine**	**Lucien**	**Lysine**	**Methionine**	**Phenylalanine**	**Threonine**	**Tryptophan**	**Tyrosine**	**Valine**
C1	68.160 ± 3.23g	145.79 ± 4.22e	987.54 ± 3.35j	199.41 ± 5.07j	149.38 ± 6.42f	376.62 ± 4.34j	280.33 ± 6.53j	49.770 ± 3.92j	287.37 ± 4.41h	363.91 ± 5.25i
C2	63.480 ± 4.14i	135.41 ± 6.41f	918.09 ± 5.06k	185.61 ± 3.01k	138.60 ± 7.51f	346.86 ± 6.41k	260.37 ± 4.41	46.310 ± 4.63k	267.18 ± 6.31i	336.61 ± 3.22j
T1	121.66 ± 5.02c	207.34 ± 2.29d	1,361.1 ± 21.7d	640.20 ± 10.03e	212.00 ± 4.32c	651.61 ± 3.14e	508.48 ± 3.33d	140.32 ± 8.95f	457.33 ± 3.53d	642.30 ± 5.04d
T2	151.18 ± 3.12b	243.48 ± 5.5b	1,584.9 ± 51.8b	869.94 ± 9.05b	248.70 ± 7.32b	805.99 ± 7.05b	636.67 ± 5.66b	187.27 ± 6.37c	551.81 ± 6.62b	793.85 ± 3.75b
T3	180.91 ± 6.33a	276.84 ± 2.35a	1,809.5 ± 62.2a	1,101.1 ± 41.9a	285.40 ± 3.53a	961.34 ± 51.4a	762.52 ± 8.37a	235.77 ± 7.58a	650.60 ± 2.77a	955.50 ± 6.57a
T4	63.920 ± 4.51h	171.09 ± 2.54e	1,044.5 ± 27.5i	409.59 ± 5.73i	176.20 ± 3.71e	482.30 ± 4.73i	312.77 ± 5.46i	97.380 ± 2.56i	326.89 ± 6.33g	451.60 ± 2.73h
T5	62.320 ± 3.01j	190.13 ± 6.26d	1,091.1 ± 66.9h	510.63 ± 3.64h	195.00 ± 7.41d	550.82 ± 7.63h	336.93 ± 6.43h	122.64 ± 8.54g	359.01 ± 3.72f	508.56 ± 6.44g
T6	61.860 ± 5.62k	208.24 ± 6.65c	1,147.7 ± 63.6g	617.39 ± 6.58f	213.80 ± 6.71c	616.36 ± 3.29f	371.87 ± 7.64g	148.05 ± 6.46e	389.44 ± 8.82e	567.16 ± 8.79e
T7	90.780 ± 1.42f	188.86 ± 8.74d	1,187.9 ± 30.7f	514.70 ± 3.29g	194.10 ± 5.82d	568.42 ± 7.37g	414.17 ± 3.83f	118.40 ± 1.55h	393.57 ± 9.93e	549.29 ± 5.38f
T8	104.50 ± 6.71e	215.64 ± 4.28b	1,323.8 ± 71.8e	678.25 ± 7.28d	221.85 ± 4.42c	676.84 ± 2.46d	486.20 ± 5.32e	153.88 ± 5.34d	456.29 ± 4.08d	653.77 ± 4.37d
T9	118.13 ± 8.23d	242.36 ± 3.66b	1,459.1 ± 71.9c	840.73 ± 4.43c	249.60 ± 8.31b	787.30 ± 4.57c	566.04 ± 3.51c	188.05 ± 3.43b	516.73 ± 3.01c	758.60 ± 7.05c
	**Alanine**	**Arginine**	**Aspartic acid**	**Glutamic acid**	**Glycine**	**Histidine**	**Proline**	**Serine**
C1	418.68 ± 8.31j	307.51 ± 6.31i	464.36 ± 9.32j	1,525.3 ± 13.44h	257.80 ± 7.42j	205.12 ± 2.41g	682.28 ± 4.43i	375.43 ± 6.01j
C2	389.55 ± 7.52k	285.91 ± 3.41j	432.69 ± 8.21k	1,412.2 ± 15.33i	238.93 ± 4.31k	191.49 ± 4.31g	635.62 ± 7.32k	350.18 ± 4.41k
T1	552.21 ± 5.65e	784.70 ± 6.09f	1,229.2 ± 15.17e	2,561.1 ± 20.54d	497.58 ± 6.45e	350.14 ± 2.63d	923.20 ± 5.67d	666.93 ± 6.35d
T2	634.00 ± 8.04b	1,024.9 ± 13.56c	1,625.9 ± 23.26b	3,128.6 ± 26.30b	626.68 ± 8.65b	433.50 ± 6.05b	1,091.8 ± 14.46b	823.24 ± 4.76b
T3	720.59 ± 9.35a	1,270.2 ± 15.38a	2,023.9 ± 29.28a	3,710.3 ± 31.64a	747.28 ± 4.33a	509.77 ± 6.46a	1,216.6 ± 18.06a	978.54 ± 3.47a
T4	457.28 ± 6.63i	602.58 ± 10.82h	731.62 ± 8.39i	1,780.0 ± 14.77g	331.35 ± 7.24i	267.46 ± 7.22f	672.72 ± 8.72j	398.77 ± 7.24i
T5	492.37 ± 4.74h	774.83 ± 9.05f	889.33 ± 7.59h	1,966.6 ± 12.97f	377.27 ± 5.57h	302.97 ± 4.62e	697.17 ± 9.36h	421.73 ± 8.73h
T6	508.88 ± 6.06f	933.01 ± 12.33d	1,037.1 ± 10.14f	2,162.8 ± 27.25e	426.38 ± 8.77f	339.69 ± 5.64d	711.03 ± 7.27g	444.53 ± 5.86g
T7	500.80 ± 7.68g	692.04 ± 11.52g	975.41 ± 12.08g	2,176.2 ± 18.30e	412.75 ± 4.09g	308.98 ± 9.83e	798.35 ± 5.69f	531.66 ± 9.44f
T8	555.97 ± 4.69d	898.38 ± 18.98e	1,247.3 ± 18.12d	2,562.4 ± 27.21d	500.92 ± 7.36d	369.33 ± 4.36c	896.96 ± 8.88e	625.77 ± 6.84e
T9	616.72 ± 3.88c	1,088.7 ± 12.46b	1,520.2 ± 21.20c	2,938.8 ± 14.70c	590.96 ± 9.66c	424.47 ± 5.75b	971.86 ± 7.49c	717.63 ± 7.45c

The means carrying the same letter in a column differed non-significantly (p > 0.05); C1, control 1; C2, control 2; T1, corn snacks supplemented with 20/100 g of soy flour; T2, corn snacks supplemented with 30/100 g of soy flour; T3, corn snacks supplemented with 40/100 g of soy flour; T4, corn snacks supplemented with 20/100 g of chickpea flour; T5, corn snacks supplemented with 30/100 g of chickpea flour; T6, corn snacks supplemented with 40/100 g of chickpea flour; T7, corn snacks supplemented with 10:10/100 g of soy and chickpea flour; T8, corn snacks supplemented with 15:15/100 g of soy and chickpea flour; T9, corn snacks supplemented with 20:20/100 g of soy and chickpea flour.

These values of the amino acid composition of the extrusion formulations were compared with the experimental results of the final extruded snacks' amino acid profile to estimate the effect of extrusion processing on amino acid retention. The percent change during extrusion for the essential and non-essential amino acids is shown in Figure 2C. The results revealed that the amino acid that is affected the most by extrusion processing is cysteine (45.1%). Essential amino acids sensitive to extrusion processing can be arranged in the following descending order, starting from most affected to least affected, i.e., lysine, phenylalanine, isoleucine, tyrosine, methionine, and valine, with reduction percentages of 5.7, 5.6, 3.78, 3.47, 2.65, and 2.55%, respectively. Leucine (0.83%) and tryptophan (0.15%) are among the essential amino acids that remained almost unaffected during extrusion processing. Among the non-essential amino acids, the most unstable is alanine (−28.52%). Similarly, as per the results of the study, non-essential amino acids could be arranged in descending order, starting from most affected to least affected, i.e., glycine, aspartic acid, serine, arginine, glutamic acid, histidine, and proline, with reduction percentages of 9.22, 6.12, 5.43, 4.98, 4.52, 2.62, and 1.57%, respectively. This increase is probably due to the inactivation of anti-nutritional compounds such as phytates and trypsin. However, heat-sensitive amino acids' levels decreased during extrusion; the operating temperature of conventional extrusion is always more than 100°C to change the moisture in the recipe to water vapors, which in turn gives extrudates their characteristic puffiness. Another reason is the Millard reaction between sugars and amino acids due to heat during extrusion. It is a leading cause of amino acid loss during extrusion. The findings of the current study were verified by the results of the previous studies. In a study, the effect of heat treatment on the retention of amino acids in infant formula was studied. The results of the study revealed that amino acid destruction during autoclaving is almost 19.5% greater than the usual preparation method that does not include heat treatment. The study reported losses in the range of 4.1–71.5%. Maximum reductions were observed in valine, glutamine, and lysine, i.e., 71.5, 60.6, and 39.2%, respectively. Overall, 28.17% of essential and 27.13% of non-essential amino acids were affected by heat treatment (18). Another study conducted on amino acid retention in canned baby foods also provided similar results on heat treatment of the amino acid profile. It was reported that there was a decrease in amino acids during heat processing; however, it also showed that isoleucine remained unaffected by the heat treatment. The percent recovery of phenylalanine, tryptophan, and tyrosine was 116, 107, and 102%, respectively, which indicates the positive effect of heat treatment on these amino acids (33, 34).

## Conclusions

Supplementing corn snacks with soy and chickpeas increased the protein and dietary fiber content of the snacks. Soy-fortified snacks were nutritionally superior to chickpea-supplemented snacks. Corn snacks, developed by using 40/100 g soy flour, showed a high content of protein (20.67 ± 0.46%), dietary fiber (18.44 ± 0.34/100 g), calcium (100.70 ± 9.21 mg/100 g), magnesium (166.92 ± 4.98 mg/100 g), potassium (1,124.4 ± 55.32 mg/100 g), and iron (17.03 ± 1.56 mg/100 g) contents. The supplementation with soy and chickpea improved balance in the amino acid profile of the corn snacks. Amino acids such as lysine (5.70%), phenylalanine (5.6%), isoleucine (3.78%), tyrosine (3.47%), methionine (2.65%), and valine (2.55%) decrease when processed by extrusion technology to produce corn snacks.

## Data availability statement

The original contributions presented in the study are included in the article/supplementary material, further inquiries can be directed to the corresponding author.

## Author contributions

F-u-HS: writing—original draft. MS: conceptualization. ZA and RS: review and editing. AA: investigation and supervision. MJ: conducting research and performing the experiments, data analysis resources provision, and formal analysis. D-e-SS: validation and verification of data. MA: data interpretation. MJA: formal analysis. All authors contributed to the article and approved the submitted version.

## References

[1] United Nations Children's Fund. Improving Child Nutrition: The Achievable Imperative for Global Progress. New York, NY: United Nations Children's Fund (2013). Available online at: https://data.unicef.org/resources/improving-child-nutrition-the-achievable-imperative-for-global-progress (accessed November 10, 2020).

[2] Shah FUH Sharif MK Bashir S Ahsan F. Role of healthy extruded snacks to mitigate malnutrition. Food Rev. Int. (2019) 35:299–323. 10.1080/87559129.2018.1542534

[3] Altaf U Hussain SZ Qadri T Iftikhar F Naseer B Rather AH. Investigation on mild extrusion cooking for development of snacks using rice and chickpea flour blends. J Food Sci Technol. (2021) 58:1143–55. 10.1007/s13197-020-04628-7 33678896 PMC7884526

[4] World Health Organization. Supplementary Foods for the Management of Moderate Acute Malnutrition in Infants and Children 6–59 Months of Age. Geneva: World Health Organization (2012). Available online at: https://www.who.int/nutrition/publications/moderate-malnutrition/9789241504423/en/ (accessed February 18, 2021).

[5] Shah FUH Sharif MK Butt MS Shahid M. Development of protein, dietary fiber, and micronutrient enriched extruded corn snacks. J Text Stud. (2017) 48:221–30. 10.1111/jtxs.12231 28573729

[6] Darnton-Hill I Nalubola R. Fortification strategies to meet micronutrient needs: sucesses and failures. Proc Nutr Soc. (2002) 61:231–41. 10.1079/PNS2002150 12133205

[7] Reshi M Shafiq F Hussain SZ Naseer B Amin T. Physicochemical properties of iron-fortified, low glycemic index (GI) barley based extruded ready-to-eat snacks developed using twin-screw extruder. J Food Process Preserv. (2020) 44:14606. 10.1111/jfpp.14606

[8] Muldabekova BZ Umirzakova GA Assangaliyeva ZR Maliktayeva PM Zheldybayeva AA Yakiyayeva MA. Nutritional evaluation of buns developed from chickpea-mung bean composite flour and sugar beet powder. Int J Food Sci. (2022) 2022:9998. 10.1155/2022/6009998 35340441 PMC8956446

[9] Hasmadi M Noorfarahzilah M Noraidah H Zainol MK Jahurul MHA. Functional properties of composite flour: a review. Food Res. (2020) 4:1820–31. 10.26656/fr.2017.4(6).419

[10] Stojceska V Ainsworth P Plunkett A Ibanoglu S. The advantage of using extrusion processing for increasing dietary fibre level in gluten-free products. Food Chem. (2010) 121:156–64. 10.1016/j.foodchem.2009.12.024

[11] Lai YC Wang SY Gao HY Nguyen KM Nguyen CH Shih MC. Physicochemical properties of starches and expression and activity of starch biosynthesis-related genes in sweet potatoes. Food Chem. (2016) 199:556–64. 10.1016/j.foodchem.2015.12.053 26776008

[12] Zia-Ul-Haq M Iqbal S Ahmad S Imran M Niaz A Bhanger MI. Nutritional and compositional study of desi chickpea (*Cicer arietinum* L.) cultivars grown in Punjab, Pakistan. Food Chem. (2007) 105:1357–63. 10.1016/j.foodchem.2007.05.004

[13] Smart Snacking. Canada's Food Guide. (2012). Available online at: http://en.wikipedia.org/wiki/Snack_food (accessed October 25, 2020).

[14] AACC. Approved Methods of American Association of Cereal Chemists, 10th ed. St. Paul, MN: The American Association of Cereal Chemists (2000).

[15] AOAC. Association of Official Analysis Chemists. Official Methods of Analysis 18th edn. Arlington, VA: AOAC Press (2012).

[16] Hernández Y Lobo MG González M. Determination of vitamin C in tropical fruits: a comparative evaluation of methods. Food Chem. (2006) 96:654–64. 10.1016/j.foodchem.2005.04.01212941557

[17] Ryynänen M Lampi AM Salo-Väänänen P Ollilainen V Piironen V. A small-scale sample preparation method with HPLC analysis for determination of tocopherols and tocotrienols in cereals. J Food Comp Anal. (2004) 17:749–65. 10.1016/j.jfca.2003.09.01423587317

[18] Adeyeye EI Afolabi EO. Amino acid composition of three different types of land snails consumed in Nigeria. Food Chem. (2004) 85:535–9. 10.1016/S0308-8146(03)00247-4

[19] Steel RGD Torrie JH Dickey DA. Principles and procedures of statistics. In: A Biometrical Approach, 3rd edn. New York, NY: McGraw HillBook Co. Inc. (1997), p. 352–399.

[20] Pérez AA Drago SR Carrara CR De Greef DM Torres RL González RJ. Extrusion cooking of a maize/soybean mixture: factors affecting expanded product characteristics and flour dispersion viscosity. J Food Eng. (2008) 87:333–40. 10.1016/j.jfoodeng.2007.12.008

[21] Cordesse R. Value of chickpea as animal feed. In:Saxena MC Cubero JI Wery J, editors. Present Status and Future Prospects of Chickpea Crop Production and Improvement in the Mediterranean Countries. Zaragoza: International Centre for Advanced Mediterranean Agronomic Studies (1990). p. 127–31. Available online at: https://om.ciheam.org/om/pdf/a09/91605019.pdf (accessed February 18, 2021).

[22] Kaisangsri N Kowalski RJ Wijesekara I Kerdchoechuen O Laohakunjit N Ganjyal GM. Carrot pomace enhances the expansion and nutritional quality of corn starch extrudates. LWT-Food Sci Technol. (2016) 68:391–9. 10.1016/j.lwt.2015.12.016

[23] Pastor-Cavada E Drago SR González RJ Juan R Pastor JE Alaiz M. Effects of the addition of wild legumes (*Lathyrus annuus* and *Lathyrus clymenum*) on the physical and nutritional properties of extruded products based on whole corn and brown rice. Food Chem. (2011) 128:961–7. 10.1016/j.foodchem.2011.03.126

[24] Sharif MK Rizvi SS Paraman I. Characterization of supercritical fluid extrusion processed rice–soy crisps fortified with micronutrients and soy protein. LWT Food Sci Technol. (2014) 56:414–20. 10.1016/j.lwt.2013.10.042

[25] Nicole M Fei HY Claver IP. Characterization of ready-to-eat composite porridge flours made by soy-maize-sorghum-wheat extrusion cooking process. Pak J Nutr. (2010) 9:171–8. 10.3923/pjn.2010.171.178

[26] Baik OD Marcotte M. Modeling the moisture diffusivity in a baking cake. J Food Eng. (2003) 56:27–36. 10.1016/S0260-8774(02)00144-9

[27] Paraman I Wagner ME Rizvi SS. Micronutrient and protein-fortified whole grain puffed rice made by supercritical fluid extrusion. J Agric Food Chem. (2012) 60:11188–94. 10.1021/jf3034804 23066826

[28] Voltarelli FA Araujo BM Moura LP Garcia A Silva CMS Junior RCV. Nutrition recovery with spirulina diet improves body growth and muscle protein of protein-restricted rats. Int J Nutr Metab. (2011) 3:22–30. 10.5897/IJNAM.9000028

[29] Riaz MN. Healthy baking with soy ingredients. Cereal Foods World. (1999) 44:136–9.

[30] Alonso R Rubio LA Muzquiz M Marzo F. The effect of extrusion cooking on mineral bioavailability in pea and kidney bean seed meals. Anim Feed Sci Technol. (2001) 94:1–13. 10.1016/S0377-8401(01)00302-9

[31] Berrios JDJ. Extrusion cooking of legumes: dry bean flours. Encyclopedia Agric Food Boil Eng. (2006) 1:1–8. 10.1081/E-EAFE-120041506

[32] Onwulata CI Konstance RP Smith PW Holsinger VH. Co-extrusion of dietary fiber and milk proteins in expanded corn products. LWT Food Sci. Technol. (2001) 34:424–9. 10.1006/fstl.2000.0742

[33] American Association of Cereal Chemists and Approved Methods Committee. Approved Methods of the American Association of Cereal Chemists, Vol. 1. Saint Paul, MN: Amer. Assn. of Cereal Chemists (2000).

[34] Saadat S Akhtar S Ismail T Sharif MK Shabbir U Ahmad N. Multilegume bar prepared from extruded legumes flour to address protein energy malnutrition. Ital J Food Sci. (2020) 32:1559. 10.14674/IJFS-1559

